# Experience-driven Predictability Does Not Influence Neural Entrainment to the Beat

**DOI:** 10.1162/JOCN.a.95

**Published:** 2026-02-01

**Authors:** Joshua D. Hoddinott, Molly J. Henry, Jessica A. Grahn

**Affiliations:** University of Western Ontario; Toronto Metropolitan University

## Abstract

Humans spontaneously synchronize movements to a perceived underlying pulse, or beat, in music. Beat perception may be indexed by the synchronization of neural oscillations to the beat, marked by increases in EEG amplitude at the beat frequency [Nozaradan, S., Peretz, I., Missal, M., & Mouraux, A. Tagging the neuronal entrainment to beat and meter. *Journal of Neuroscience*, *31*, 10234–10240, 2011]. Indeed, neural synchronization to the beat appears stronger for strong-beat than non-beat rhythms [Tal, I., Large, E. W., Rabinovitch, E., Wei, Y., Schroeder, C. E., Poeppel, D., et al. Neural entrainment to the beat: The “missing-pulse” phenomenon. *Journal of Neuroscience*, *37*, 6331–6341, 2017] and may underlie the generation of an internal representation of beat. However, because we are exposed disproportionately to strong-beat rhythms (e.g., most Western music) in the environment, comparisons of neural responses to strong-beat and non-beat rhythms may be confounded by relative differences in familiarity. Here, we dissociated beat-related and familiarity-related neural responses by comparing EEG amplitudes during the perception of strong-beat and non-beat rhythms that were either novel or made familiar through training. First, we recorded EEG from participants while they listened to a set of strong-beat, weak-beat, and non-beat rhythms. Then, they were trained on half of the rhythms over four behavioral sessions by listening to and tapping along with them, such that half of the rhythms were familiar by the end of training. Finally, EEG responses to the full rhythm set (half now familiar, half still unfamiliar) were recorded posttraining. Results show no effect of training on EEG amplitude at beat or stimulus-related frequencies and little evidence of familiarity-driven changes in EEG amplitude for weak- and non-beat rhythms. This suggests that oscillatory entrainment to the beat is not driven by familiarity and therefore likely reflects beat processing.

## INTRODUCTION

Across cultures, listening to music elicits spontaneous synchronous behavior, such as foot tapping, aligned to the underlying pulse, or beat, in music (London, 2012). Synchrony also occurs in neural activity, which tracks rhythm, beat, and other acoustic features of music (Cheng, Creel, & Iversen, 2022; Flaten, Marshall, Dittrich, & Trainor, 2022; Weineck, Wen, & Henry, 2022; Gilmore & Russo, 2020; Lenc, Keller, Varlet, & Nozaradan, 2018). Neural activity tends to be more synchronized when music is highly familiar (Weineck et al., 2022), suggesting that human experience with music may enhance synchrony. Because humans listen to music often, rhythms that have a beat are heard much more frequently than irregular non-beat rhythms and are therefore more familiar (we define “familiarity” as memory of a stimulus arising from repeated exposure). Thus, even carefully designed studies of neural synchronization to rhythms may be confounded by the relative differences in familiarity between regular strong-beat rhythms and control rhythms that are both irregular and unfamiliar. The current experiment was designed to dissociate whether the enhanced neural synchronization to strong-beat rhythms was related to familiarity-based predictability or to the perception of the beat. If familiarity alone is responsible for neural synchronization, then highly familiar non-beat rhythms should elicit neural responses similar to strong-beat rhythms. If, however, differences in neural synchronization remain between even highly familiar non-beat rhythms and novel strong-beat rhythms, then the enhanced neural synchronization response to strong-beat rhythms is likely to be driven by beat-related processing.

Neural synchrony observed in response to musical rhythm may represent neural entrainment. Neural entrainment is elicited by a stimulus, such as a flickering dot or a beeping tone, presented at a regular rate, and that rate is similar to the rate of endogenous neural oscillations (Lakatos, Gross, & Thut, 2019)—naturally occurring periodic fluctuations of excitation and inhibition in neural populations (Henry & Obleser, 2012; Lakatos et al., 2005). Endogenous neural oscillations become “entrained” by aligning their phase and frequency to that of the regular stimulus (Lakatos et al., 2019), measured by increased amplitude peaks in EEG frequency spectra (Lakatos, Karmos, Mehta, Ulbert, & Schroeder, 2008; Lakatos et al., 2005). For example, when a listener hears a 2-Hz metronome, their EEG amplitude spectrum will have a peak at 2 Hz, marking an enhanced amplitude fluctuation in the EEG signal at that frequency (Nozaradan, Peretz, Missal, & Mouraux, 2011). Neural entrainment facilitates perception and behavior, such as increased speech pattern intelligibility (Zoefel & VanRullen, 2016), or greater target detection accuracy during the excitable phase of neural oscillations (Henry, Herrmann, & Obleser, 2014, 2016; Henry & Obleser, 2012; Neuling, Rach, Wagner, Wolters, & Herrmann, 2012; Ng, Schroeder, & Kayser, 2012). Even processes that are top–down, such as attention allocation, can influence neural entrainment measures (Lakatos et al., 2019), suggesting that neural entrainment may not simply reflect bottom–up tracking of external stimulation but may also represent important “internal” processes, such as imposing a beat onto music (Cheng et al., 2022; Nozaradan, Schönwiesner, Caron-Desrochers, & Lehmann, 2016; Fujioka, Ross, & Trainor, 2015; Nozaradan, Peretz, & Mouraux, 2012; Nozaradan et al., 2011; Bolton, 1894).

Indeed, in rhythms with a beat, EEG amplitude is increased at the beat rate, beyond that present in the stimulus spectrum (Tal et al., 2017; Nozaradan et al., 2012). When listening to a metronome, participants who imagine a strong–weak pattern of accents (i.e., feeling the beat every two notes) exhibit increased EEG amplitude at the imagined rate, in addition to the metronome rate. Furthermore, when the same participants imagine a strong–weak–weak pattern (i.e., feeling the beat every three notes), a peak in EEG amplitude is observed at this ternary beat rate, even though the metronome stimulus is identical between binary and ternary conditions (Nozaradan et al., 2011). Even without explicit instruction to impose a beat, when primed with a binary (4/4) or ternary (3/4) metric context before hearing a rhythm with ambiguous meter (i.e., a rhythm in which the beat can be felt at either binary or ternary rates), EEG amplitude peaks are observed at the rate induced by the preceding context and not the irrelevant rate that was not primed (Nave, Hannon, & Snyder, 2022). Similarly, when listening to a rhythm that begins with a clear beat but becomes more syncopated over time (shifting to a weak-beat rhythm), EEG amplitude peaks are maintained during syncopation. However, when the same rhythm is played in reverse—with high syncopation (weak beat) at the beginning with increasing regularity over time, the weak-beat portion no longer shows peaks. This suggests that the EEG amplitude is not driven entirely by the stimulus itself but is also driven by maintaining the beat internally (Lenc, Keller, Varlet, & Nozaradan, 2020b). Together, these previous findings suggest that imposing a beat or inferring a beat from a stimulus that affords it increases neural entrainment.

The role of neural entrainment in rhythm and beat perception may be related to prediction. Various theories of neural entrainment suggest that oscillatory activity serves a predictive function (Lakatos et al., 2019). For example, the dynamic attending theory (Large & Jones, 1999) describes neural entrainment as an efficient, dynamic allocation of attention, with heightened attention at times of expected stimulus input. The neural resonance theory (NRT) posits that neural oscillators may reflect musical beat and meter via high-frequency bursts of activity entrained to external rhythmic input (Large & Snyder, 2009). Though NRT is not as descriptive of the function of oscillatory activity, it describes how high-frequency neuronal bursts persist in the absence of expected rhythmic input (e.g., a missing on-beat note), which relies on prediction. However, it remains unclear whether prediction alone is responsible for enhanced neural entrainment to the beat. Predictions can be made based on perceived regularity—such as the predictable interval of a metronome—or they can be made based on learned experience—such as the interval between a traffic light turning from yellow to red. Regular rhythms often have sounds occurring at regular intervals (e.g., a sound occurs every second), making future sound events inherently predictable. In contrast, stimuli that are heard often, even if irregular, can be implicitly learned (Batterink, Paller, & Reber, 2019; Pavlidou & Bogaerts, 2019), and predictions can be made based on the implicit memory or implicit familiarity. Strong-beat rhythms are predictable in both ways: To give rise to a beat, note-to-note onsets are highly regular, and strong-beat rhythms are highly prevalent in music, making them very familiar. Therefore, it is unclear whether beat perception drives entrainment, or if our familiarity with strong-beat rhythms drives entrainment through experience-based predictability.

There is mixed evidence for the influence of experience-driven predictability on neural entrainment to rhythm. In infants, experience with music predicts the degree of neural entrainment to the beat—infants who attended music classes had larger EEG peaks at the beat frequency than infants who did not attend (Cirelli, Spinelli, Nozaradan, & Trainor, 2016), suggesting greater familiarity with rhythm influences neural entrainment. However, it is also possible that attending music classes improved infant beat perception. In adults, neural synchronization with music is greater for familiar than unfamiliar music—EEG time series is more correlated with the spectral flux (the velocity of spectral changes over the course of a song) of familiar than unfamiliar songs (Weineck et al., 2022), suggesting that one's experience with a song can alter the relationship between brain responses and spectral acoustic information. Finally, one experiment compared neural responses to pattern-based predictions, rhythms in which predictability was increased through repetition, and beat-based predictions, where predictability was based on having a regular beat, using similar rhythmic stimuli as the current study (Bouwer, Fahrenfort, Millard, Kloosterman, & Slagter, 2022). Pattern-based expectations were elicited by repeating a short, rhythmic pattern (that had no clear beat) several times in a trial. Beat-based expectations were elicited by using nonrepeating patterns that elicit a strong beat percept (similar to those used in Bouwer, Burgoyne, Odijk, Honing, & Grahn, 2018; Grahn & Brett, 2007; Povel & Essens, 1985). Entrainment was measured in the silent period after each trial, with the hypothesis that oscillatory entrainment should persist even in the absence of stimulation. Beat-based, but not pattern-based, expectations elicited persistent neural entrainment in the silent period, suggesting that neural entrainment only occurs in the presence of an underlying beat, but not for irregular rhythms made predictable through repetition. One limitation, however, is that the short induction period for the pattern-based expectations may not have sufficiently overcome the longer-term experience-driven predictability of beat-based rhythms arising from familiarity with music. Furthermore, use of musically implausible control rhythms (i.e., non-beat rhythms) may disrupt any predictions at the rate of the beat—because the non-beat rhythms were designed to elicit no sense of beat, repetitive presentations could not elicit beat-based predictions (an absent beat cannot be predicted). Here, we familiarized participants with strong-beat, non-beat, and “weak-beat” rhythms, the latter being highly syncopated, making beat perception possible, but difficult. The weak-beat rhythms are a useful addition to test whether neural entrainment is correlated with experience-driven predictability only when a beat can be felt. Furthermore, because weak-beat rhythms are not as irregular as the non-beat rhythms, they are more likely to be successfully learned within an experiment.

Despite some evidence that neural entrainment is specific to beat-based rhythms (Bouwer et al., 2022), neural entrainment experiments have not yet directly manipulated familiarity with strong-beat, weak-beat, and non-beat rhythms. In the current experiment, we tested the influence of familiarity on neural entrainment to the beat by employing a behavioral training paradigm, familiarizing participants with strong-beat, weak-beat, and non-beat rhythms. We measured neural entrainment using EEG as people listened to 24 unique rhythms in two EEG sessions: pretraining and posttraining. Between the EEG sessions, participants completed four behavioral training sessions to become familiar with half (12) of the rhythms. Thus, in the posttraining EEG session, half of the rhythms were novel (heard only during the pretraining session), and half of the rhythms were familiar (heard, synchronized to, and reproduced several times over the four training sessions). If neural entrainment to the beat is driven by familiarity, we would expect amplitude at the beat frequency to be equal across strong-, weak-, and non-beat conditions for familiar rhythms. Additionally, weak-beat and non-beat amplitude should be significantly increased posttraining relative to pretraining. Alternatively, if greater amplitude at the beat frequency is not driven by familiarity, but rather by neural entrainment to a beat, we would expect no changes in beat frequency amplitude from pretraining to posttraining EEG sessions for any rhythms and significantly greater beat frequency amplitude for strong-beat than weak- and non-beat rhythms across all sessions. There is one caveat to this prediction: Because weak-beat rhythms are structured in a way that a beat *can* be felt (though much less easily than in strong-beat rhythms), exposure may increase the likelihood of beat perception in this condition. If this occurred, we would expect that beat-frequency amplitude would also increase from pre- to posttraining sessions, but for the weak-beat condition only. Additionally, because the non-beat rhythms have little energy at the beat frequency, it is possible that even familiarity-based neural entrainment will be absent at the beat frequency in this condition. Thus, we additionally tested neural entrainment at the peak stimulus frequency (the frequency of greatest amplitude in the stimulus spectra) for each rhythm condition. Following the same hypothesis as above: Familiarity-based neural entrainment will be indicated by increased posttraining EEG amplitudes at stimulus-related frequencies.

## METHODS

### Participants

Twenty-one English-speaking subjects (11 female) participated in the experiment. One participant was removed for having under 15% of EEG trials remaining after artifact rejection in the posttraining session. With the remaining 20 participant sample, we were able to detect medium to large effects (Cohen's *d* > 0.66) at 80% statistical power. Participants were on average 22.56 (*SD* = 4.84) years of age and had some musical training (*M* = 6.44 years, *SD* = 6.63). All participants reported normal hearing and had no history of neurological illness. Participants were recruited via poster advertisements posted at the University of Western Ontario. Participation was voluntary, and volunteers were compensated $5 per half-hour. The study was approved by the Nonmedical Research Ethics Board.

### Stimuli

Auditory rhythms were generated for each of three beat strength conditions, strong-beat, weak-beat, and non-beat rhythms, taken from stimuli used in previous work (Grahn & Brett, 2007). Each condition included eight unique rhythms, with a total of 24 rhythms used across the experiment. The 24 rhythms were organized into four counterbalanced sets, such that each set had four rhythms in each beat strength condition and was completely unique to one other set (see Table 1 for a list of stimulus sets). For example, Sets 1 and 2 contain unique sequences relative to each other, and Set 1 shares two rhythms per beat strength condition with Sets 3 and 4. Rhythms were created using 500-Hz sine tones with 8-ms onset/offset ramps. Each tone “filled” most of the duration of each interval, ending 40 msec short of the interonset duration to leave a 40-msec silent “gap” to demarcate the tone onsets (as in previous work, Grahn & Brett, 2007). Thus, the interval durations for each rhythm were the tone interonset intervals. Rhythms included six or seven intervals, and each was about 3 sec long. A final tone, the length of the shortest interval in the rhythm, was appended to the end of all sequences, such that the end of the final interval was demarcated by the final tone's onset.

**Table 1.** Rhythmic Stimuli

*Stimulus Set*	*Strong Beat*	*Weak Beat*	*Non-beat*
Set 1	1 1 1 1 4 3 1	1 4 1 1 3 1 1	1 3.6 1 4 1 1 1
	2 1 1 2 2 3 1	1 1 3 2 2 1 2	1.4 1 1.4 3.6 1.4 1 1
	1 1 2 3 1 4	1 2 2 1 4 2	1.4 1 4 1.4 1.4 1
	2 2 1 3 3 1	2 3 1 1 2 3	1 3.6 1.4 3.6 1.4 1

Set 2	2 1 1 3 1 1 3	2 3 3 1 1 1 1	3.6 1 1 3.6 1 1.4 1
	3 1 4 1 1 1 1	2 1 4 1 2 1 1	3.6 1.4 1.4 1 1 1 1.4
	3 1 2 2 1 3	3 2 3 2 1 1	1.4 1.4 1 1.4 4 1
	3 1 1 3 2 2	4 1 2 2 1 2	4 1.4 1 3.6 1 1

Set 3	1 1 1 1 4 3 1	2 1 4 1 2 1 1	1 3.6 1 4 1 1 1
	2 1 1 3 1 1 3	1 1 3 2 2 1 2	3.6 1 1 3.6 1 1.4 1
	1 1 2 3 1 4	1 2 2 1 4 2	1 3.6 1.4 3.6 1.4 1
	3 1 1 3 2 2	3 2 3 2 1 1	1.4 1.4 1 1.4 4 1

Set 4	2 1 1 2 2 3 1	2 3 3 1 1 1 1	3.6 1.4 1.4 1 1 1 1.4
	3 1 4 1 1 1 1	1 4 1 1 3 1 1	1.4 1 1.4 3.6 1.4 1 1
	2 2 1 3 3 1	4 1 2 2 1 2	4 1.4 1 3.6 1 1
	3 1 2 2 1 3	2 3 1 1 2 3	1.4 1 4 1.4 1.4 1

1 = 250 msec; 2 = 500 msec; 3 = 750 msec; 4 = 1000 msec.

Strong- and weak-beat rhythms were generated using integer ratio intervals, such that interval durations were multiples of the shortest or “1” interval (i.e., 1:2:3:4). Non-beat rhythms replaced the “2” and “3” intervals with noninteger ratios (1:1.4:3.6:4). For each rhythm, the shortest interval (i.e., the “1” interval) was 250 msec. Only one tempo was used such that the phase of the EEG response could be aligned across stimuli, allowing averaging of the trials. Strong-beat rhythms were arranged such that perceptual accents occurred at evenly spaced time points—at the beginning of each group of four units, marking the location of the beat (Povel & Essens, 1985). Weak-beat and non-beat rhythms were arranged such that perceptual accents did not occur at evenly spaced time points, making the beat more difficult to detect (Grahn & Brett, 2007). Additionally, non-beat rhythms were created using noninteger ratio intervals (i.e., 1:1.4:3.6:4), which eliminated regularity in the rhythm, such that no beat existed.

Because EEG time–frequency analysis requires relatively long trials, ∼18-sec versions of the rhythms were generated. These long rhythms were created by looping the 3-sec rhythm 6 times. For each loop, the final “1” interval downbeat was replaced by the first interval of the next iteration of the rhythm. Only the sixth presentation included a final downbeat to signify the end of the rhythm. For example, the rhythm 3 1 2 2 1 3 would repeat: 3 1 2 2 1 3 3 1 2 2 1 3 […] 3 1 2 2 1 3 1.

### Procedure

**Figure 1.**
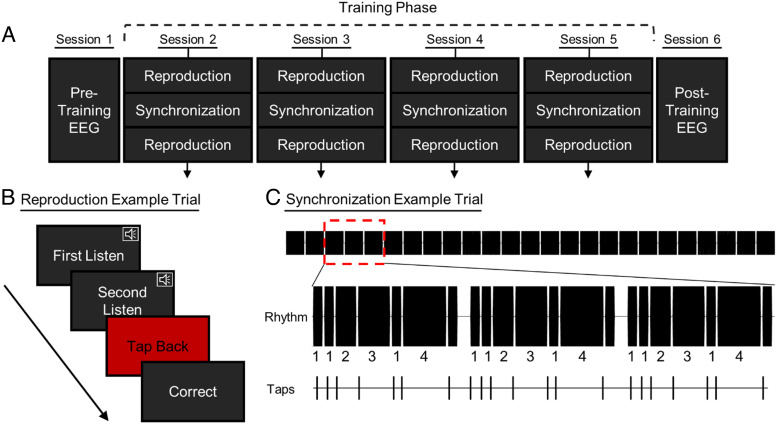
Experimental procedure and training tasks. (A) Procedure and task order across the six experimental sessions. Subjects attended an EEG session pretraining and again posttraining. Behavioral training occurred in Sessions 2–5 and included two tasks: Rhythm reproduction (two blocks per session) and rhythm synchronization (one block per session). (B) Schematic depiction of a rhythm reproduction trial. Participants tapped (in silence) after hearing two repetitions of a rhythm. (C) Stimulus depiction for one trial of the rhythm synchronization task. Rhythms repeated 25 times per trial while participants tapped along to the sounds. Integers indicate relative durations of intervals in the sample trial. The rhythm is shown as an audio wave form (top: 25 repetitions; middle: zoomed-in view of three repetitions at red square), with black indicating sound and white indicating silence. Taps as depicted in time are a hypothetical example of how a participant might tap during the synchronization task.

The experiment included six separate sessions (Figure 1), each occurring at least 24 hr (or more) after the previous session. In the first and final sessions, EEG was recorded as participants listened to all 24 long rhythms and performed a target detection task (described below). In the four middle sessions, no EEG was collected. Instead, participants were trained on half (12) of the rhythms; four rhythms from each of the three beat strength conditions. Tasks used in the training sessions are described below.

#### EEG Sessions

EEG setup began after the experiment was explained verbally, and participants read and signed a letter of information. Data collection took place in a dimly lit, sound-attenuated booth. Experimenters monitored EEG acquisition and task progression via a laptop outside of the booth. Before starting the tasks, a hearing threshold test was used to establish an appropriate sound volume set at 55-dB sensation level for each participant independently. During EEG acquisition, the different rhythms were played in a pseudorandom order, while a white fixation dot appeared on a black background. Participants performed a target detection task. Afterward, a beat-tapping and rating task was performed. Participants also completed a self-reported engagement questionnaire at the end of each EEG session.

#### Tasks: Target Detection

To ensure engagement with the stimuli during EEG acquisition, participants detected targets. Between one and four tones on a trial were amplitude modulated at 40 Hz and 100% amplitude depth (target tones). Participants counted the number of target tones silently and responded via keyboard whether they heard one, two, three, or four target tones when prompted at the end of each trial. After a response was entered, the next trial began.

Each EEG session included six blocks (∼8 min each) of the target detection task. In each block, all 24 rhythms were presented. Rhythms of the same beat strength condition were presented consecutively within the block. However, the order of the rhythms within a beat strength condition was random, and the order of beat strength conditions was random across blocks. Before the first block, a practice trial was presented.

#### Tasks: Beat Tapping/Rating

After the EEG equipment was removed, participants performed beat-tapping and rating tasks. Here, participants again heard all 24 rhythms (long, 18-sec versions). Participants were instructed to tap to the beat as each rhythm played. When the rhythm stopped, the screen displayed three consecutive questions: (1) “How well do you think you tapped the beat?” (2) “How strongly did you feel the beat BEFORE you started tapping?” (3) “How familiar were you with the rhythm?” Rating questions were always presented in this order. Each question required a response from 1 to 100 via keyboard input. A rating of 1 was low on each scale (i.e., poor tapping, weak beat perception, not very familiar). The rating scale appeared on the screen with each question.

#### Tasks: Engagement Questionnaire

Finally, at the end of each EEG session, participants completed a questionnaire that probed for attentiveness and understanding of the target detection task during EEG acquisition.

#### Behavioral Sessions

In Sessions 2–5, rhythm reproduction and synchronization tasks were used to familiarize participants with half (12) of the rhythms. Participants also performed the Beat Alignment Test (BAT) in one of the training sessions (Hsu, Ready, & Grahn, 2022; Müllensiefen, Gingras, Musil, & Stewart, 2014). Each behavioral session included two blocks of rhythm reproduction. Between each rhythm reproduction block, rhythm synchronization was implemented. All tasks in the behavioral session used short (3-sec) rhythms that were not looped.

#### Tasks: Rhythm Reproduction

On each trial, participants heard a randomly selected rhythm twice (ISI = 1100 msec). After the second presentation, a response window with a red screen with black letters indicated “Tap Back.” The response window lasted for the duration of the rhythm, plus 4 sec. Participants reproduced the rhythm by tapping a computer key. After the response window, a screen (black, white letters) presented either “Correct” or “Incorrect” feedback for the trial. If each interval in a rhythm was reproduced within 15% of its true duration (e.g., a 500-msec interval could be accurately demarcated by taps separated by 425–575 msec), the trial was considered correct. Any interval outside of this threshold resulted in an incorrect trial. Trials with too many or too few taps were also considered incorrect. Participants were made aware of these rules on the first training session and reminded, if necessary, throughout training. Regardless of performance, each rhythm was repeated on two consecutive trials. After each trial, a screen indicated whether the same rhythm would be repeated or a new rhythm would be presented. To limit discouragement, the first block of reproduction in Training Session 1 used a more liberal 20% accuracy threshold. Participants were made aware of this difference.

#### Tasks: Rhythm Synchronization

Rhythm synchronization was used to further improve learning of the rhythms, as synchronization provides the participant the opportunity to hear the interval onset time relative to their own tap time, and it allows many presentations of each rhythm. On each trial, each rhythm was repeated 25 times. The interrhythm interval was the 40-msec gap, as also done for each interval in the rhythm, plus 250 msec of silence; thus, the silent gaps were not aligned with the beat. However, the consistency of the interrhythm interval enabled participants to anticipate the onset of the next repetition of the rhythm. Participants tapped the tone onsets on a contact microphone as the rhythms played. The stimulus rhythm and tapped response waveforms were recorded simultaneously in separate channels in Audacity (as in Jacoby & McDermott, 2017).

Before the task, participants were instructed to notice whether they tapped too early or too late for each of the tones and adjust tapping as the rhythms repeated. No feedback was given for performance on this task. As this task was lengthy, each trial was initiated by the participant, and participants were encouraged to take breaks between trials if they felt their attention was waning.

#### Tasks: BAT

The BAT (Hsu et al., 2022; Müllensiefen et al., 2014) measured participants' ability to produce and perceive a beat when listening to short music clips. In the production test, participants listened to music clips and tapped the beat on a computer key in time with the beat that they perceived. After each trial, participants rated their familiarity with the stimulus.

In the beat perception test, participants listened to the same music clips, but this time paired with an isochronous series of tones that was timed to be either on or off the beat. Participants decided whether the tone was on or off the beat and indicated via button press as soon as they knew their response. BAT production and perception tests were presented in the same order for each participant.

### EEG Acquisition

EEG was recorded using a 64-channel BioSemi system. Data were acquired at 1024-Hz sampling frequency.

#### Preprocessing

All EEG data were preprocessed using Fieldtrip (Oostenveld, Fries, Maris, & Schoffelen, 2010) and custom MATLAB (MathWorks, Inc.) functions. EEG data were re-referenced to the average of left and right external mastoid electrodes. Data were filtered between 0.1 Hz (high-pass) and 30 Hz (low-pass) using windowed-sinc finite impulse response. Variance of whole-trial epochs was visually inspected, and any problematic channels or trials were manually removed. The remaining trials were passed on to an independent components analysis. Components were inspected visually, and noise/artifact components (e.g., blinks, lateral eye movements, muscle activity) were rejected. Next, any channels removed from visual inspection were interpolated by averaging signal from neighboring electrodes within 40 mm of the missing channel. Finally, trials in which any channel fluctuated more than 300 μV were removed from further analysis.

### Analysis

#### EEG

For each EEG session, trials from each condition were averaged together in the time domain to produce six mean time series (strong, weak, non-beat, with familiar and novel versions of each) at each of the 64 EEG channels. Time-domain averaging was used to better isolate activity that is phase-locked to the trials (i.e., oscillatory entrainment) from non-phase-locked activity (Nozaradan et al., 2011). These time series were then transformed into periodic amplitude and phase using the fast Fourier transform (FFT), with a frequency resolution of 0.0125 Hz. The amplitude spectra were then averaged across the 64 channels, giving six frequency spectra for the pretraining and six for posttraining EEG sessions for each participant. To insure that session-specific aperiodic noise did not influence the analysis, a 1/frequency function between 0.5 and 6 Hz was fitted and then subtracted from each participant's frequency spectra for each session and beat strength conditions independently (using custom code, based on methods from the FOOOF toolbox; Donoghue et al., 2020). Removing the aperiodic noise “flattens” the frequency spectra but allows periodic signals to remain as peaks above 0 μV. Thus, session-to-session effects cannot be explained by differences in the magnitude or shape of aperiodic noise between sessions.

#### Frequencies of Interest

Analyses of EEG amplitude were carried out on three frequencies of interest. The main research question focused on the 1-Hz beat frequency—the rate of the beat across strong- and weak-beat rhythms. Two other frequencies of interest reflected stimulus-driven responses (i.e., the average rate of tones in the rhythms). To determine the stimulus-driven frequency for each beat strength condition, we identified the largest peak in the averaged stimulus amplitude spectrum. For each rhythm, an FFT was performed on the absolute Hilbert transform of the sound (i.e., the envelope of the waveform). The resulting amplitude frequency spectra were averaged for rhythms of each beat strength condition, providing three average frequency spectra (strong, weak, and non-beat). For the strong-beat and weak-beat rhythms, the peak stimulus frequency was 4 Hz. For the non-beat rhythms, the peak stimulus frequency was 3.34 Hz.

#### Statistical Analysis

EEG amplitudes at each of the frequencies of interest were entered into 2 (session: pretraining, posttraining) × 2 (rhythm set: trained, untrained) × 3 (beat strength: Strong, weak, non) repeated-measures ANOVAs. All significant main effects and interactions were explored with follow-up *t* tests.

In addition to frequentist statistics, the same 2 × 2 × 3 repeated-measures ANOVA was analyzed using Bayesian statistics. Bayesian statistics are a useful analytic approach as evidence for the null hypothesis can be quantified, as opposed to rejecting or failing to reject the null. Bayesian statistics tests whether the prior likelihood of a model (i.e., the chance-level likelihood of selecting a model to fit the data; P(M)) is increased after considering the data (P(M|Data)). If a model fits the data well, the likelihood of selecting the model will increase from chance, [posterior likelihood P(M|Data) > prior likelihood P(M)]. In the case of an ANOVA, all combinations of main effects, interactions, and a null hypothesis (no effects) were entered as models that were equally likely to explain the data. Therefore, the prior likelihood of selecting any of these models is 1/# of models (Table 2, P(M) column). Next, the likelihood of each model is redistributed across all models, based on how well the model fits the data (Table 2, P(M|Data) column)—models that fit the data increase in likelihood, and models that poorly fit the data decrease or do not change from the prior. The BF_M_ represents the change from prior to posterior likelihood of selecting a model, with larger BF meaning more support for the model fitting the data: In general, 3 < BF < 10 suggests moderate support for a model, and BF > 10 suggests strong support (Stefan, Gronau, Schönbrodt, & Wagenmakers, 2019).

#### Rhythm Reproduction

Performance on the rhythm reproduction task was assessed using average proportional error. Average proportional error is the unsigned (absolute) difference between a reproduced interval and the target stimulus interval, divided by the stimulus interval (|Reproduced − Target|/Target). The average proportional error takes the mean proportional error across all intervals in a trial. Perfect performance would result in 0 average proportional error. Trials with too many or too few taps were removed from this analysis, as it is not clear which tap corresponds to which stimulus interval.

## RESULTS

### Rhythm Reproduction Performance

**Figure 2.**
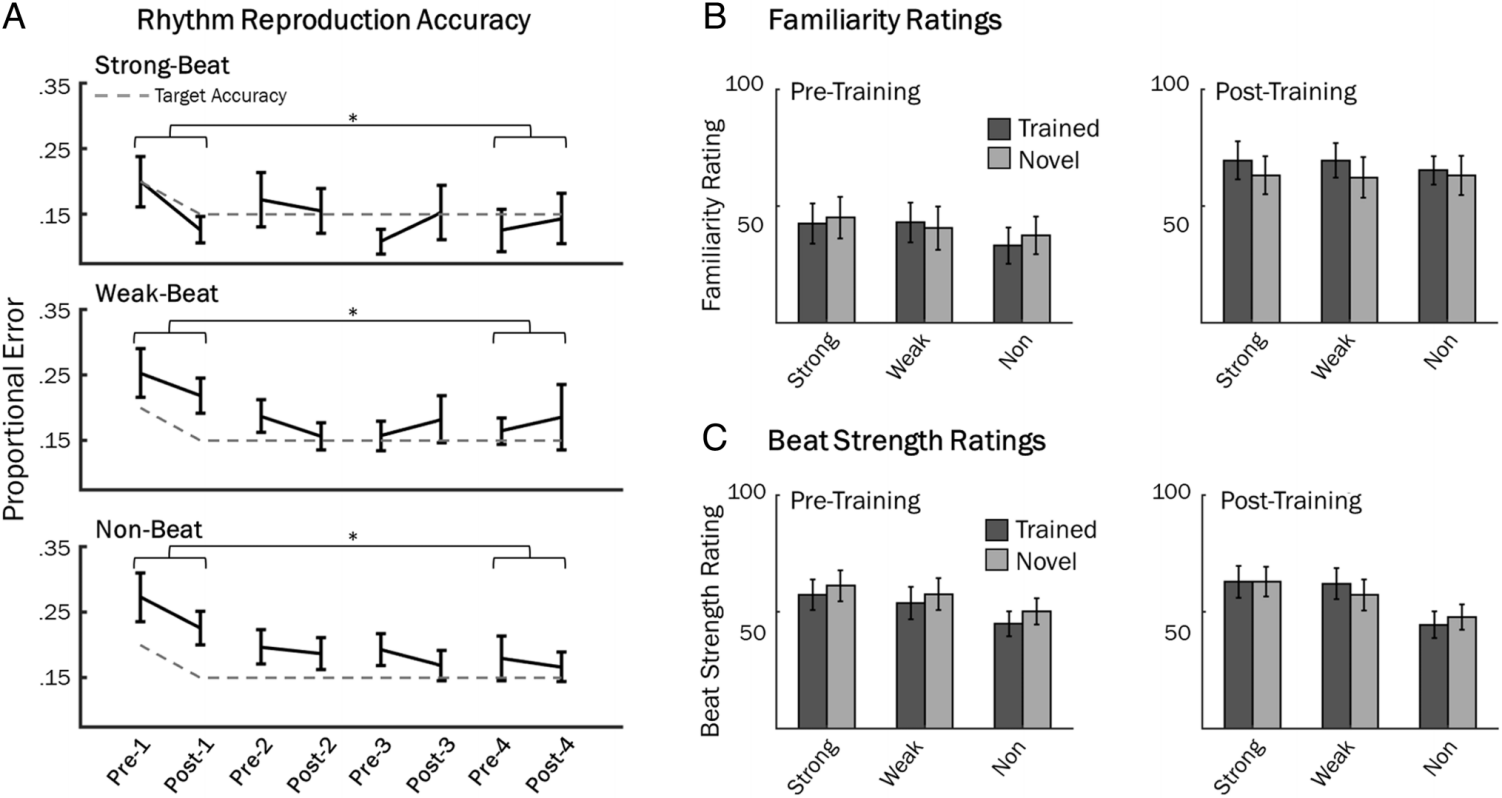
Behavioral results. (A) Average proportional error on the rhythm reproduction task across training blocks. (B) Ratings of familiarity for trained and novel rhythms in pre- and posttraining EEG sessions. (C) Ratings of beat strength for trained and untrained rhythms in pre- and posttraining sessions. Error bars represent *SEM* in all plots.

Performance on the rhythm reproduction task is shown in Figure 2. Performance across behavioral training sessions was evaluated using the average proportional error. Average error was calculated independently for each beat strength condition and averaged across the two blocks of the rhythm reproduction task for each session. This resulted in four session-averaged error scores for each beat strength condition. To determine whether accuracy on the rhythm reproduction task differed between beat strength conditions, average error from the first session and last session were entered into a 2 (session: first, last) × 3 (beat strength: strong, weak, non-beat) repeated-measures ANOVA. One participant was removed from this analysis, as they did not tap the correct number of times in one condition in Session 2, resulting in no data. The 2 × 3 ANOVA revealed a significant main effect of beat strength, *F*(2, 36) = 15.16, *p* < .001, and a significant main effect of session, *F*(1, 18) = 24.11, *p* < .001. No significant interaction was found, *F*(2, 36) = 0.78, *p* = .47. Follow-up *t* tests were used to investigate the main effects. For beat strength, strong-beat rhythms (*M* = 0.16, *SD* = 0.15) were performed more accurately than weak-beat rhythms (*M* = 0.21, *SD* = 0.13), *t*(19) = −2.97, *p* = .008, and non-beat rhythms (*M* = 0.22, *SD* = 0.12), *t*(19) = −3.95, *p* < .001. Performance on weak and non-beat rhythms was not significantly different *t*(19) = −1.13, *p* = .27. The session main effect was driven by poorer performance in Session 1 (*M* = 0.23, *SD* = 0.12), than Session 4 (*M* = 0.16, *SD* = 0.14), showing that training improved performance on the rhythm reproduction task.

To test whether the “rate” of learning depended on beat strength, a linear fit was conducted across the four chronologically ordered sessions, and each participant's rate of performance improvement during training was summarized by the slope of average error scores across the four sessions for each beat strength condition. Average error slopes were entered into a one-way (beat strength: strong, weak, non-beat) repeated-measures ANOVA. No significant effect of beat strength was found, *F*(2, 34) = 1.5, *p* = .24, suggesting performance improved at the same rate across sessions, regardless of beat strength.

The four-session training paradigm was designed to equalize familiarity between strong-beat rhythms and weak- and non-beat rhythms. To test whether this occurred, the average proportional error across the final training session was entered into independent paired samples *t* tests. At the end of training, strong-beat rhythms (*M* = 0.13, *SD* = 0.15) were still performed more accurately than weak-beat (*M* = 0.18, *SD* = 0.15), *t*(19) = 4.19, *p* < .001, and non-beat rhythms (*M* = 0.18, *SD* = 0.13), *t*(19) = 2.96, *p* = .008. Accuracy on weak-beat and non-beat rhythms did not differ at the end of training, *t*(19) = 0.30, *p* = .77. Though performance was not equated by end of training (mainly because accuracy on strong-beat rhythms increased simultaneously), reproduction accuracy for weak-beat and non-beat rhythms in the “final” training session did not statistically differ from strong-beat rhythms in the “first” training session (*p*s > .26). Furthermore, Bayesian *t* tests revealed anecdotal evidence for the null hypothesis when comparing posttraining weak-beat and pretraining strong-beat performance (BF_10_ = 0.43) and moderate evidence for the null when comparing posttraining non-beat and pretraining strong-beat performance (BF_10_ = 0.25). Thus, the training paradigm succeeded in matching weak-beat and non-beat rhythm performance after training to that of strong-beat rhythms before training. Thus, if familiarity is driving neural entrainment, then all rhythms should show increased amplitude at the beat frequency from pre- to posttraining, and EEG amplitudes should be similar for pretraining strong-beat rhythms and posttraining weak- and non-beat rhythms.

### Beat Strength and Familiarity Ratings

In pretraining and posttraining EEG sessions, participants rated each rhythm on perceived beat strength and familiarity. Due to a technical error, data from only 15 participants were available for both sessions. To test whether training influenced perceived beat strength, the beat strength ratings were entered into a 2 (session) × 2 (rhythm set: trained, untrained) × 3 (beat strength: strong, weak, non-beat) repeated-measures ANOVA (Figure 2). This analysis revealed no main effect for session, *F*(1, 14) = 0.17, *p* = .69, or rhythm set, *F*(1, 14) = 3.04, *p* = .10, suggesting that familiarity did not influence perceived beat strength. A significant main effect of beat strength was found, *F*(2, 28) = 7.64, *p* = .002, and followed up with paired sample *t* tests. Overall, strong-beat rhythms (*M* = 54.81, *SD* = 24.42) were rated significantly higher than weak-beat rhythms (*M* = 51.12, *SD* = 24.43), *t*(14) = 2.24, *p* = .042, and non-beat rhythms (*M* = 41.55, *SD* = 18.88), *t*(14) = 2.92, *p* = .034. Weak-beat rhythms were rated significantly higher than non-beat rhythms, *t*(14) = 2.61, *p* = .041. No significant interactions were found: Rhythm Set × Beat Strength, *F*(2, 28) = 1.98, *p* = .16; Rhythm Set × Session, *F*(1, 14) = 3.09, *p* = .10; Beat Strength × Session, *F*(2, 28) = 2.29, *p* = .12; Rhythm Set × Beat Strength × Session, *F*(2, 28) = 1.60, *p* = .22.

Due to technical error, several participants were missing familiarity ratings from the pretraining session. Familiarity ratings from 11 participants were collected in both pretraining and posttraining sessions and entered into a 2 (session) × 2 (rhythm set: trained, untrained) × 3 (beat strength: strong, weak, non-beat) repeated-measures ANOVA. A significant main effect of session was found, *F*(2, 10) = 8.70, *p* = .015, and a significant main effect of beat strength was found, *F*(1.14, 20) = 5.08, *p* = .041. There was no main effect of rhythm set, *F*(1, 10) = 0.91, *p* = .36. Additionally, a Session × Rhythm Set interaction was marginally significant, *F*(1, 10) = 4.21, *p* = .067. All other interactions were not significant (*p*s > .17). The main effects of session and beat strength were explored using post hoc *t* tests. Across all rhythm types, the posttraining session (*M* = 68.27, *SD* = 24.44) had significantly greater familiarity ratings than the pretraining session (*M* = 43.47, *SD* = 26.80), *t*(10) = 2.95, *p* = .015. The main effect of beat strength was driven by higher familiarity ratings for the strong-beat rhythms (*M* = 58.13, *SD* = 22.87) than the non-beat rhythms (*M* = 52.37, *SD* = 20.17), *t*(10) = 2.99, *p* = .022, and by higher familiarity ratings for the weak-beat rhythms (*M* = 57.11, *SD* = 22.42) than the non-beat rhythms, *t*(10) = 2.46, *p* = .046. Importantly, strong- and weak-beat rhythms were not rated differently on familiarity, *t*(10) = 0.53, *p* = .60. Finally, we explored the marginal Session × Rhythm Set interaction with post hoc *t* tests. Overall, the trained rhythm set was rated significantly more familiar in the posttraining session (*M* = 70.32, *SD* = 21.82), than the same trained rhythm set in pretraining (*M* = 42.74, *SD* = 26.16), *t*(10) = 3.24, *p* = .05. In the pretraining session, familiarity ratings for the trained rhythm set and untrained rhythm set (*M* = 44.21, *SD* = 27.69) did not significantly differ, *t*(10) = 0.76, *p* = .46. In the posttraining session, differences in familiarity between the trained rhythm set and untrained rhythm set (*M* = 66.22, *SD* = 27.31) approached significance, *t*(10) = 2.12, *p* = .09. Somewhat surprisingly, familiarity ratings for the untrained rhythm set were marginally greater in the posttraining session, compared with the same untrained rhythm set in the pretraining session, *t*(10) = 2.59, *p* = .08, perhaps due to participants including familiarity with the task, and with the pitch of the tones in their familiarity ratings. Bayesian analysis of the posttraining familiarity ratings revealed moderate evidence for the null hypothesis for trained strong-beat versus weak-beat rhythms (BF_10_ = 0.28) and anecdotal evidence for the null for trained strong-beat versus non-beat rhythms (BF_10_ = 0.64), suggesting trained rhythms were equally familiar between beat strength conditions in the posttraining session, though the effect may be weak due to low sample size (*n* = 13).

### Training-related Changes to Neural Entrainment at 1 Hz

**Figure 3.**
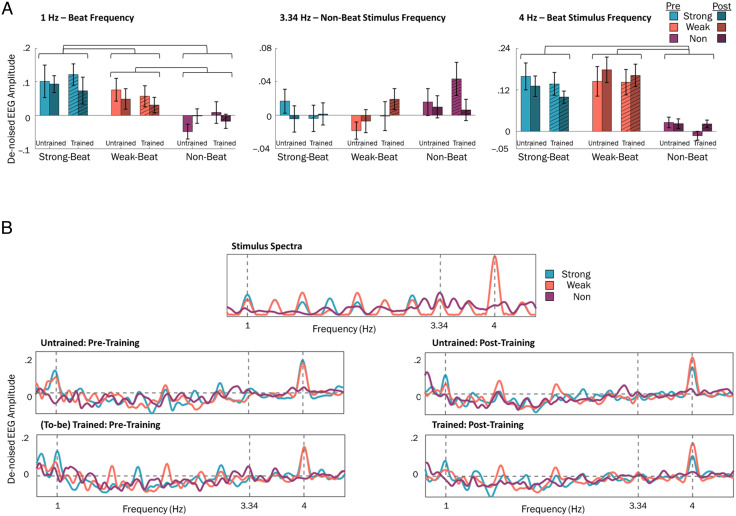
EEG amplitude spectra were not influenced by behavioral training. Top: De-noised EEG amplitude at the beat frequency (1 Hz; left), non-beat stimulus frequency (3.34 Hz; middle), and strong/weak-beat stimulus frequency (4 Hz; right). Light colors represent pretraining, and dark colors represent posttraining. Amplitudes for the trained stimulus set marked by hashed bars. Error bars show *SEM*. Bottom: De-noised EEG amplitude spectra from 0.75 to 4.5 Hz for each session (left: pretraining, right: posttraining) and stimulus set (top: untrained, bottom: trained).

To evaluate whether training influenced neural entrainment to the beat, we examined spectral amplitude at the beat frequency (1 Hz) for each session, beat strength condition, and for novel/familiar rhythm sets. Mean EEG spectra are shown in Figure 3, and individual data are shown in Figure 5. Differences in beat frequency amplitudes were evaluated using a 2 (session: pretraining, posttraining) × 3 (beat strength: strong, weak, non-beat) × 2 (rhythm set: trained, untrained) repeated-measures ANOVA. There were no significant main effects of session, *F*(1, 19) = 0.507, *p* = .49, or familiarity, *F*(1, 19) = 0.002, *p* = .97, suggesting training did not alter spectral amplitude at the beat frequency. A significant main effect of beat strength, *F*(2, 38) = 14.38, *p* < .001, was found and investigated using follow-up *t* tests. Across the experiment, strong-beat rhythms (*M* = 0.097, *SD* = 0.089) elicited greater amplitude at the beat frequency than weak-beat rhythms (*M* = 0.053, *SD* = 0.055), *t*(19) = 2.18, *p* = .042, and non-beat rhythms (*M* = −0.015, *SD* = 0.050), *t*(19) = 4.44, *p* < .001. Weak-beat rhythms also elicited greater amplitude at the beat frequency than non-beat rhythms, *t*(19) = 4.04, *p* < .001. Overall, these findings suggest that, as expected, strong-beat rhythms elicit the greatest neural entrainment at the beat frequency, followed by weak-beat rhythms and non-beat rhythms. Training had no impact on neural entrainment to the beat.

The same 2 × 2 × 3 ANOVA was performed again using Bayesian statistics, which evaluates how accurately each main effect and interaction (and a null effect) models the data. Here, the BF_M_ represents the posterior likelihood of selecting a model as the model of best fit, with larger BF meaning more support for the model fitting the data. In general, 3 < BF < 10 suggests moderate support for a model, and BF > 10 suggests strong support (Stefan et al., 2019). In our case, the best-fitting model was the main effect of beat strength (Table 2, Row 1), which had strong support, BF_M_ = 25.33. The following Bayes factors (BF_10_) reflect the ratio of evidence for the alternative model over the best-fitting model (the main effect of beat strength). For example, in Table 2, Row 2, including a main effect of both beat strength and session is more likely than chance after considering the data (BF_M_ = 3.71), but it is only 29% as likely to fit the data compared with a model with only a main effect of beat strength. In other words, the model with only beat strength is 3.44 [1/0.29 = 3.44] times more likely to explain the data than a model with both beat strength and session effects. In alignment with the frequentist statistical test findings described above, the main effect of beat strength was the best fitting model for the data. Evidence for main effects of session (BF_10_ = 5.27 × 10^−4^) and rhythm set (BF_10_ = 3.79 × 10^−4^) was very low.

**Table 2.** Bayesian Model Comparisons for EEG Amplitude at 1 Hz

*Models*	*P(M)*	*P(M|Data)*	*BF_M_*	*BF_10_*	*Error %*
Beat	0.05	0.59	25.33	1.00	
Beat + Session	0.05	0.17	3.71	0.29	2.10
Beat + Rhythm Set	0.05	0.12	2.54	0.21	2.15
Beat + Session + Rhythm Set	0.05	0.034	0.65	0.06	2.81
Beat + Session + Beat * Session	0.05	0.03	0.52	0.05	2.26
Beat + Rhythm Set + Beat * Rhythm Set	0.05	0.02	0.39	0.04	2.51
Beat + Session + Rhythm Set + Session * Rhythm Set	0.05	0.02	0.28	0.03	3.77
Beat + Session + Rhythm Set + Beat * Rhythm Set	0.05	0.006	0.11	0.01	2.68
Beat + Session + Rhythm Set + Beat * Session	0.05	0.006	0.11	0.01	2.58
Beat + Session + Rhythm Set + Beat * Rhythm Set + Session * Rhythm Set	0.05	0.003	0.05	0.005	3.30
Beat + Session + Rhythm Set + Beat * Session + Session * Rhythm Set	0.05	0.003	0.05	0.004	3.02
Null model (incl. subject and random slopes)	0.05	0.001	0.02	0.002	1.21
Beat + Session + Rhythm Set + Beat * Session + Beat * Rhythm Set	0.05	0.001	0.02	0.002	2.78
Beat + Session + Rhythm Set + Beat * Session + Beat * Rhythm Set + Session * Rhythm Set	0.05	4.62e^−4^	0.008	7.90e^−4^	5.48
Session	0.05	3.08e^−4^	0.006	5.27e^−4^	1.89
Rhythm Set	0.05	2.22e^−4^	0.004	3.79e^−4^	2.02
Beat + Session + Rhythm Set + Beat * Session + Beat * Rhythm Set + Session * Rhythm Set + Beat * Session * Rhythm Set	0.05	1.17e^−4^	0.002	2.00e^−4^	5.65
Session + Rhythm Set	0.05	6.50e^−5^	0.001	1.11e^−4^	2.87
Session + Rhythm Set + Session * Rhythm Set	0.05	3.00e^−5^	5.39e^−4^	5.13e^−5^	7.33

P(M) = prior probability of each model; P(M|data) = probability of each model, taking the data into account; BF_m_ = Bayes factor of each model; BF_10_ = Bayes factor of the current model over the best model (beat strength). All models include subject, and random slopes for all repeated-measures factors.

Some of the models that included combined effects had high evidence for fitting the data, even if not the winning model. To explore the unique contributions of each main effect and interaction, we used Bayesian statistics to quantify the change in likelihood of an effect being included in the best model after considering the data. The likelihood of each effect being included in a model of good fit is reported in Table 3. Prior (P(incl)) and posterior (P(incl)|data) probabilities of including an effect are calculated from the linear combination of prior (P(M)) and posterior (P(M)|data) probabilities across all models that include the effect (e.g., P(incl) for the beat strength effect is the sum of P(M) values in Table 2 for all models including beat strength). This analysis revealed that the beat strength factor is very likely to explain the data (BF_incl_ = 209.99), while all other main effects and interactions are very unlikely (all BF_incl_ < 0.13).

**Table 3.** Bayesian Analysis of Effects for EEG Amplitude at 1 Hz

*Effects*	*P(incl)*	*P(excl)*	*P(incl|data)*	*P(excl|data)*	*BF_incl_*
Beat strength	0.74	0.26	0.998	0.002	209.99
Session	0.74	0.26	0.27	0.73	0.13
Rhythm set	0.74	0.26	0.22	0.79	0.10
Beat Strength * Session	0.32	0.68	0.04	0.96	0.09
Beat Strength * Rhythm Set	0.32	0.68	0.03	0.97	0.07
Session * Rhythm Set	0.32	0.68	0.02	0.98	0.05
Beat Strength * Session * Rhythm Set	0.05	0.95	1.17e^−4^	1.00	0.002

P(incl) = chance-level probability of including the effect in a best-fit model; P(excl) = chance-level probability of excluding the effect from the best-fit model; P(incl|data) = updated probability of including the effect given its fit to the data across all models; P(excl|data) = updated probability of excluding the effect given its fit to the data across all models; BF_incl_ = the change from chance to updated inclusion probability.

**Figure 4.**
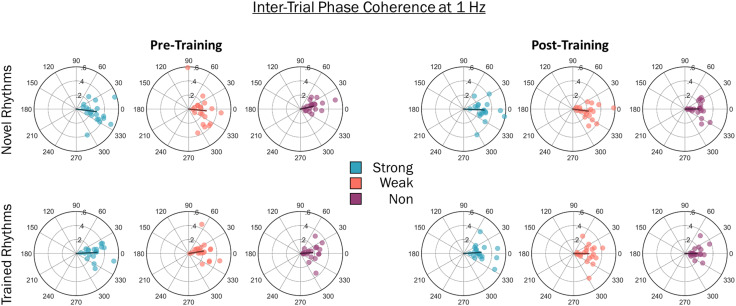
Intertrial phase coherence at 1 Hz for the three beat strength conditions in each EEG session (left column: pretraining, right column: posttraining) and for each rhythm set (top row: untrained; bottom row: trained). On each circular plot, the length of the vector represents the mean resultant vector length, *r*, and the angle of the vector represents the mean angle across subjects. Dots represent the mean vector length and angle for individual subjects. As in the EEG amplitude analysis, intertrial phase coherence was greatest for the strong-beat rhythms, compared with weak- and non-beat rhythms, and weak-beat rhythms elicited greater intertrial phase coherence than non-beat rhythms. Intertrial phase coherence was unaltered by training.

Finally, oscillatory entrainment is often marked by phase synchrony—peaks in the EEG amplitude temporally aligned with stimulus input (Henry & Obleser, 2012). To test for phase synchrony, the phase of EEG activity at 1 Hz was taken from single-trial FFTs for each beat strength condition, session, and rhythm set. Using the Circular Statistics Toolbox (Berens, 2009), the intertrial phase coherence (ITPC) was calculated for each beat strength condition, session, and rhythm set. On each trial for a given participant, an FFT was applied at and then averaged across all EEG channels, resulting in a complex frequency spectrum (including phase and amplitude information) for each trial. For each beat strength condition and rhythm set, the resultant vector length of the angle of the EEG data at 1 Hz was calculated and is shown in Figure 4. Large resultant vector lengths indicate high ITPC or consistent phase across trials. The resultant vector lengths were entered into a 2 (session: pretraining, posttraining) × 3 (beat strength: strong, weak, non-beat) × 2 (familiarity: familiar, novel) repeated-measures ANOVA. Only a significant main effect of beat strength was found, *F*(2, 38) = 14.84, *p* < .001. There was no significant main effect of session, *F*(1, 19) = 0.35, *p* = .56, or rhythm set, *F*(1, 19) = 2.09, *p* = .16. No significant interactions were found (all *p*s > .55). The main effect of beat strength was explored with post hoc *t* tests. Collapsed across rhythm sets and session, ITPC at 1 Hz was greatest for strong-beat rhythms (*M* = 0.29, *SD* = 0.07) compared with weak-beat rhythms (*M* = 0.23, *SD* = 0.07), *t*(19) = 3.11, *p* = .007, and compared with non-beat rhythms (*M* = 19, *SD* = 0.05), *t*(19) = 5.43, *p* < .001). Weak-beat rhythms had significantly greater ITPC than non-beat rhythms, *t*(19) = 2.3, *p* = .03 (Figure 4).

**Figure 5.**
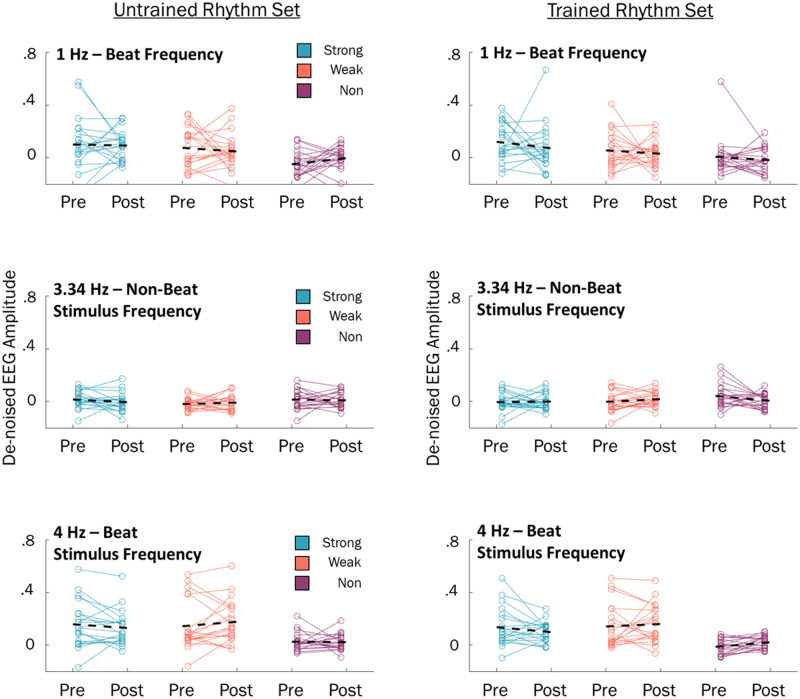
EEG amplitude at each tested frequency for the untrained (left column) and trained (right column) rhythm sets during pre- and posttraining EEG sessions. Individual subjects plotted as colored lines; group mean indicated by black line.

### Training-related Changes to Neural Entrainment at 4 Hz

In addition to the beat frequency, we tested the peak stimulus frequency from each beat strength condition using an identical 2 (session) × 2 (stimulus set) × 3 (beat strength) repeated-measures ANOVA. The peak stimulus frequency for strong- and weak-beat rhythms was 4 Hz. As in the beat frequency, amplitude at 4 Hz was unaffected by training, revealing no effect of session, *F*(1, 19) = 0.039, *p* = .85, or rhythm set, *F*(1, 19) = 3.43, *p* = .08. However, a main effect of beat strength was found, *F*(1.29, 24.49) = 15.11, *p* < .001. Follow-up *t* tests revealed that strong-beat rhythms (*M* = 0.13, *SD* = 0.12) had greater amplitude at 4 Hz than non-beat rhythms (*M* = 0.014, *SD* = 0.031), *t*(19) = 4.24, *p* < .001. Similarly, weak-beat rhythms (*M* = 0.16, *SD* = 0.14) had greater amplitude at 4 Hz than non-beat rhythms, *t*(19) = 5.15, *p* < .001. Amplitude at 4 Hz did not differ between strong-beat and weak-beat rhythms, *t*(19) = −0.91, *p* = .37. As expected, strong-beat and weak-beat rhythms elicited greatest amplitude at stimulus-relevant frequencies, though this amplitude was unaffected by familiarity training.

### Training-related Changes to Neural Entrainment at 3.34 Hz

As the non-beat stimulus spectra revealed greatest amplitude at 3.34 Hz, an independent 2 (session: pretraining, posttraining) × 2 (rhythm set: Trained, untrained) × 3 (beat strength: strong, weak, non-beat) repeated-measures ANOVA was performed on EEG amplitude at this frequency. Similar to the 1- and 4-Hz analyses, behavioral training had no effect on EEG amplitude at stimulus-relevant frequencies, revealing no main effects for session, *F*(1, 19) = 0.21, *p* = .65, or rhythm set, *F*(1, 19) = 1.33, *p* = .26. No main effect of beat strength was found, *F*(2, 38) = 2.18, *p* = .13, though numerically non-beat rhythms had the greatest amplitude at this frequency (Figure 3). There were no significant interactions found at this frequency: Rhythm Set × Beat Strength, *F*(2, 38) = 1.21, *p* = .31; Rhythm Set × Session, *F*(1, 19) = 0.01, *p* = .94; Beat Strength × Session, *F*(2, 38) = 1.52, *p* = .23; Rhythm Set × Beat Strength × Session, *F*(2, 38) = 1.77, *p* = .19.

### Evaluation of Aperiodic Noise Correction

As intended, the 1/f aperiodic noise correction kept periodic signals intact: EEG amplitudes at beat and stimulus frequencies remained positive. However, visual inspection of the EEG spectra revealed negative amplitudes at a portion of other frequencies, indicating possible overcorrection. To determine whether noise was systematically different between rhythm conditions or EEG sessions, we entered the coefficients *a* (the *y*-intercept of the curve) and *b* (the decay factor) from the estimated formula *a* × *b*^*x*^ into independent 3 (beat strength) × 2 (session) × 2 (rhythm set; trained, novel) repeated-measures ANOVAs. In both tests, no significant main effects or interactions were found (all *p*s > .16). This suggests that the aperiodic noise correction was consistent across experimental conditions and did not overcorrect in some of the conditions or sessions compared with others, which could drive or mask effects in the above analyses. Thus, comparison and interpretation of noise-corrected EEG amplitudes across the experiment is appropriate.

### Musicianship and Behavioral Correlations at 1 Hz

To test for a relationship between amplitude at 1 Hz and the magnitude of experience-driven predictability, Pearson correlations were calculated for EEG amplitude in pretraining and posttraining EEG sessions with self-reported years of music playing, the average reproduction error in the first and last training sessions, and for BAT production asynchrony and coefficient of variation. There were no significant correlations between 1-Hz amplitude and musicianship (*p*s > .33), rhythm reproduction performance (*p*s > .084), or BAT production (*p*s > .10). As in the mean comparisons, this suggests that neural entrainment to the beat is not related to explicit learning of rhythm. Similarly, correlations between each participant's mean beat strength ratings and EEG amplitude at 1 Hz for the training rhythms in pre- and posttraining sessions were not significant (*p*s > .14), suggesting that perceived beat strength did not necessarily drive neural entrainment to the beat.

## DISCUSSION

This experiment was designed to disentangle whether experience-driven predictability or predictability based on the presence of a beat drives neural entrainment to rhythm. Participants were trained over four behavioral sessions to accurately reproduce half of the rhythms they heard in a pretraining and posttraining EEG session. By equalizing familiarity with strong-beat, weak-beat, and non-beat rhythms, we were able to test whether familiarity alone could drive neural entrainment at the beat frequency (1 Hz). No differences were found in EEG amplitude or intertrial phase coherence between pretraining and posttraining for either the trained or untrained rhythms in any beat strength condition. In fact, numerically (but not significantly), EEG amplitude was lower in the posttraining session. Furthermore, in the posttraining session, trained rhythms did not elicit different amplitude or phase from the untrained rhythms, suggesting that session-to-session reliability of EEG signals did not obscure an effect of training. Across both EEG sessions, strong-beat rhythms elicited the greatest EEG amplitude at the beat frequency, compared with weak- and non-beat rhythms, replicating previous findings of neural entrainment to a clear beat (Bouwer et al., 2022; Gilmore & Russo, 2020; Lenc, Keller, Varlet, & Nozaradan, 2020a; Lenc et al., 2018, 2020b; Cameron et al., 2019; Tal et al., 2017). Overall, this suggests that neural entrainment at the beat frequency is not driven by experience-driven predictability.

In addition to the beat frequency, we also compared the peak frequency in the averaged stimulus spectra for the strong- and weak-beat (4 Hz) and the non-beat condition (3.34 Hz; see Figure 3B). Responses at the peak stimulus frequencies likely reflect neural responses to the tone onset rate, rather than an internal representation of beat. Similar to responses at the beat frequency, these stimulus-driven responses were unaffected by training, with no changes to EEG amplitude or phase coherence from pretraining to posttraining for the trained rhythms, nor differences between trained versus untrained rhythms posttraining.

The absence of a training effect on neural entrainment to rhythm and beat is not explained by insufficient behavioral training. Performance accuracy on the reproduction task improved at the same rate for all beat strength conditions, and all conditions were reproduced significantly more accurately at the end of training than at the beginning of training, suggesting that rhythms in all beat strength conditions were well learned and highly predictable. Furthermore, by training's end, weak-beat and non-beat rhythms were performed as accurately as strong-beat rhythms in the first training session, suggesting the real-world experience-driven advantage of strong-beat rhythms was successfully matched by training. Despite these behavioral improvements, we found no corresponding change in EEG amplitude or phase coherence, suggesting that neural entrainment to beat and rhythm is not related to mechanisms of rhythm reproduction performance that would be altered throughout training, such as prediction error encoding, motor planning, or explicit memorization of rhythms. Rather, neural entrainment to beat and rhythm appears tied to rhythm perception and may instead be related to the inherent predictability of strong-beat rhythms (due to temporal regularity) or the generation of an internal representation of beat.

Our findings support recent evidence that neural entrainment to the beat does not correspond to rhythm predictability. Previous findings show that neural entrainment is elicited only by beat-based rhythms and not repetitive (i.e., highly predictable) non-beat rhythms (Bouwer et al., 2022). Because that study manipulated predictability using within-trial repetition, the mechanism relied on STM, which likely differs from that underlying experience-driven predictability of beat-based rhythms. Our paradigm trained participants to memorize and tap strong-, weak-, and non-beat rhythms over several sessions, ensuring that each rhythm was learned, rather than stored only in STM. Despite our intensive training paradigm, predictability did not account for neural entrainment to the beat—entrainment at the beat frequency was unique to rhythms in which the beat was very clear. We additionally tested whether neural entrainment to intermediate beat strength rhythms (i.e., the weak-beat condition) was altered by experience-driven predictability. The weak-beat condition allowed us to test whether relatively unpredictable, but beat-plausible, rhythms elicit enhanced neural entrainment posttraining. However, the null effect of EEG amplitude at the beat frequency for all three beat strength conditions confirms that the experience-driven predictability does not account for neural entrainment to the beat in strong-, weak-, or non-beat rhythms. Furthermore, the null effects in this experiment are robust: Bayesian statistics showed the best model of the data included only beat strength as a factor and models including an effect of training poorly explained the data. In a study using ecologically valid stimuli, neural synchronization with the spectral flux of music depended on both the participants' familiarity with the stimulus and the ease with which participants could tap the beat (Weineck et al., 2022). Songs that were more familiar and had clearer beat resulted in the greatest correlation between the EEG signal and the stimulus' spectral flux (measured using temporal response functions; Crosse, Di Liberto, Bednar, & Lalor, 2016). Thus, as found in the current study, neural synchronization to music correlated with beat strength, but unlike our findings, familiarity also played a role. The discrepancy between current findings and previous work (Weineck et al., 2022) may suggest that familiarity impacts neural synchrony to sound when there are many stimulus features to predict, such as in real music, but for reduced-feature stimuli, such as the highly controlled simple rhythms used here, familiarity is not enough to induce synchrony.

Our findings partially conflict with developmental work in rhythm and beat perception. Infants who have more experience with music tend to have greater amplitude at beat frequencies than infants with less experience (Cirelli et al., 2016). Unlike the current findings, this suggests that neural entrainment to the beat is partially driven by experience with music. However, in the previous study, familiarity with the rhythms themselves was not manipulated, and experience was approximated by time spent in music classes. Additionally, because this study was performed on infants, it is possible that experience with music catalyzes the development of neural entrainment to the beat but does not necessarily drive entrainment in adults. That is, during infanthood, exposure may initiate the neural entrainment mechanism that underpins beat perception, resulting in differences between infants with and without musical exposure. However, in adulthood when the neural entrainment mechanism is well established, exposure does not influence the “strength” of neural entrainment—the magnitude of EEG amplitudes at the beat rate is not influenced by exposure. The data here support this interpretation, as we found no correlations between years of music practice and EEG amplitude at the beat frequency, suggesting that ecologically valid experience with music does not dictate the magnitude of oscillatory activity at the beat frequency.

Overall, our data suggest that experience-driven predictability does not account for neural entrainment to the underlying beat in rhythm. However, this does not imply that prediction is unnecessary or unrelated to neural entrainment. In general, perceiving a beat requires predicting upcoming beat locations, and the neural response to this process, though not altered by familiarity, likely reflects or encompasses some predictive process. Importantly, our data show that this process is unique to rhythms with an underlying beat and is not imposed on irregular non-beat rhythms, regardless of predictability.

Corresponding author: Joshua D. Hoddinott, Psychology Department, University of Western Ontario, London, Ontario, Canada; Centre for Brain and Mind, University of Western Ontario, London, Ontario, Canada, e-mail: jhoddin@uwo.ca.

## Data Availability Statement

Data and analysis scripts are available upon request. Supplemental Material can be accessed on this article's homepage: https://doi.org/10.1162/JOCN.a.95.

## Author Contributions

Joshua D. Hoddinott: Conceptualization; Data curation; Formal analysis; Methodology; Visualization; Writing—Original draft; Writing—Review & editing. Molly J. Henry: Formal analysis; Methodology; Writing—Original draft; Writing—Review & editing. Jessica A. Grahn: Conceptualization; Funding acquisition; Methodology; Supervision; Writing—Original draft; Writing—Review & editing.

## Funding Information

This research was supported by an NSERC Discovery Grant (RGPIN-2016-05834) and NSERC Steacie Fellowship (566202-2021-SMFSU) to J. A. G., as well as by NSERC-CREATE in Complex Dynamics, and NSERC Postgraduate Scholarship to J. D. H.

## Diversity in Citation Practices

Retrospective analysis of the citations in every article published in this journal from 2010 to 2021 reveals a persistent pattern of gender imbalance: Although the proportions of authorship teams (categorized by estimated gender identification of first author/last author) publishing in the *Journal of Cognitive Neuroscience* (*JoCN*) during this period were M(an)/M = .407, W(oman)/M = .32, M/W = .115, and W/W = .159, the comparable proportions for the articles that these authorship teams cited were M/M = .549, W/M = .257, M/W = .109, and W/W = .085 (Postle and Fulvio, *JoCN*, 34:1, pp. 1–3). Consequently, *JoCN* encourages all authors to consider gender balance explicitly when selecting which articles to cite and gives them the opportunity to report their article's gender citation balance.

## Supplementary Material


