# Supplementary figures and images for: Experience-driven Predictability Does Not Influence Neural Entrainment to the Beat
Source: J Cogn Neurosci. 2026 Feb 1;38(2):406–21. doi: 10.1162/JOCN.a.95 (PMC12829885; doi:10.1162/JOCN.a.95)

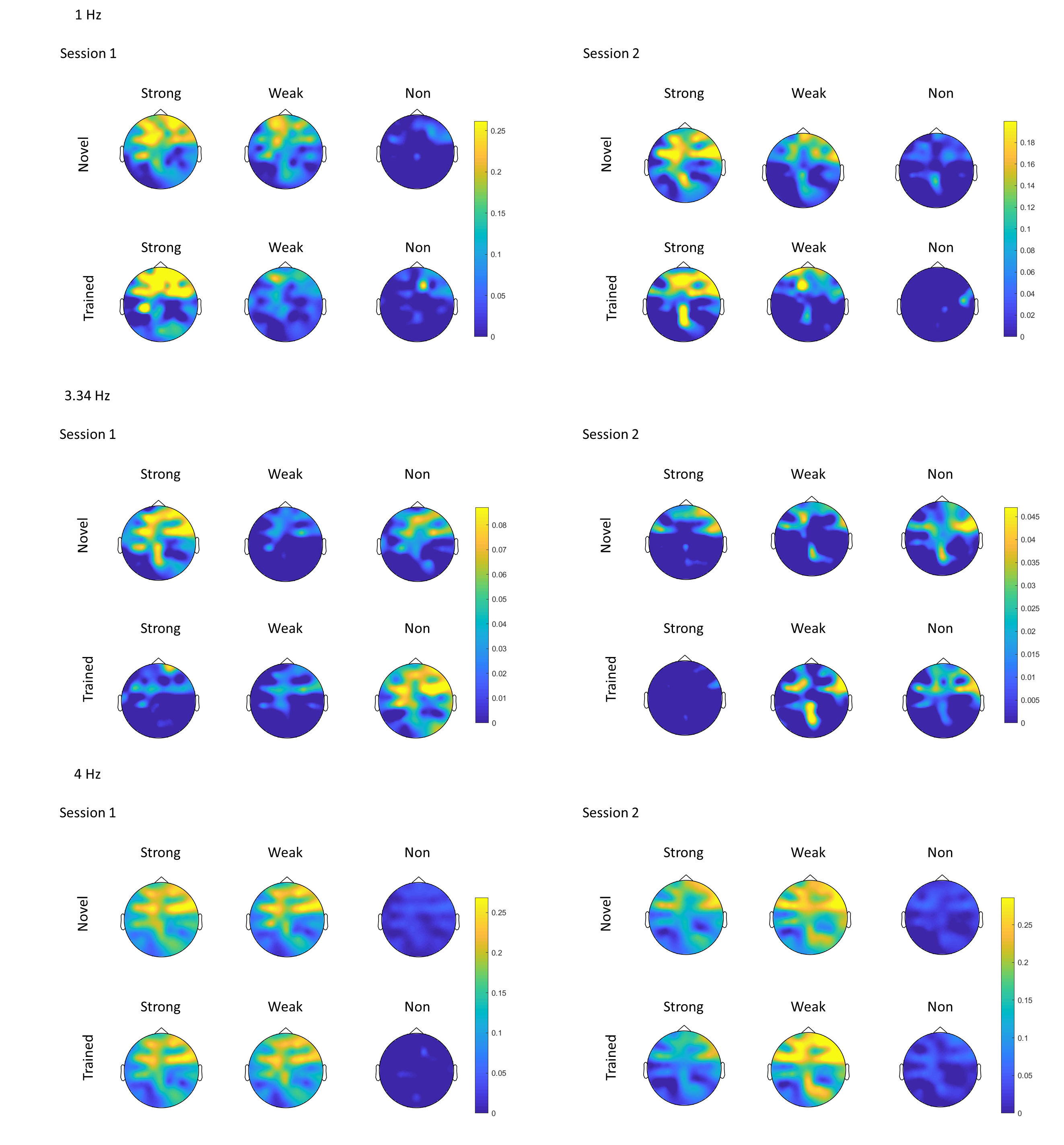


**Supplemental Figure 1.** Topographies of EEG amplitude for 1, 3.34, and 4 Hz frequencies.

Supplement: Supplementary file 1 [file jocn-38-2-406-s001.docx]
